# Say farewell to bland regression reporting: Three forest plot variations for visualizing linear models

**DOI:** 10.1371/journal.pone.0297033

**Published:** 2024-02-02

**Authors:** Jonathan Fries, Sandra Oberleiter, Jakob Pietschnig

**Affiliations:** Department of Developmental and Educational Psychology, Faculty of Psychology, University of Vienna, Vienna, Austria; University of Agder: Universitetet i Agder, NORWAY

## Abstract

Regression ranks among the most popular statistical analysis methods across many research areas, including psychology. Typically, regression coefficients are displayed in tables. While this mode of presentation is information-dense, extensive tables can be cumbersome to read and difficult to interpret. Here, we introduce three novel visualizations for reporting regression results. Our methods allow researchers to arrange large numbers of regression models in a single plot. Using regression results from real-world as well as simulated data, we demonstrate the transformations which are necessary to produce the required data structure and how to subsequently plot the results. The proposed methods provide visually appealing ways to report regression results efficiently and intuitively. Potential applications range from visual screening in the model selection stage to formal reporting in research papers. The procedure is fully reproducible using the provided code and can be executed via free-of-charge, open-source software routines in R.

## Introduction

### Reporting and visualizing regression models

Regression is one of the most widely used methods of statistical data analysis across many scientific fields [1–3], and can also be counted among the standard repertoire of statistical methods in psychology research [4, 5]. There are many variations of the concept, but they are essentially all used to predict values of one variable based on the values of another variable [6].

In its simplest form, a regression model is composed of a criterion variable (also known as dependent variable, response variable, or outcome) and a predictor variable [7]. According to the widely adopted guidelines by the American Psychological Association, researchers should at least report the test statistic, *p*-values, and confidence intervals for each predictor, as well as the coefficient of determination *R*^*2*^ for the overall model when reporting regression models in research papers [8]. However, including regression weights (i.e., the change in the dependent variable when the predictor increases by one unit) or standardized regression coefficients (i.e., the number of standard deviations the dependent variable changes when the predictor increases by one standard deviation) is strongly recommended as they are crucial in helping readers evaluate the meaningfulness of a predictor regarding its effect size [9].

Owing to the amount of numerical information, regression analyses are commonly reported in the form of tables [e.g., 10]. In many cases, such tables are useful because they can hold large amounts of information. However, with increasing number of reported models and corresponding parameters, tables tend to become tedious to read and difficult to interpret [11]. Researchers often resort to appending large regression tables to their manuscripts as supplementary materials [e.g., 12] because they consume a lot of space–a coveted resource in scientific journals [13].

**Fig 1:**
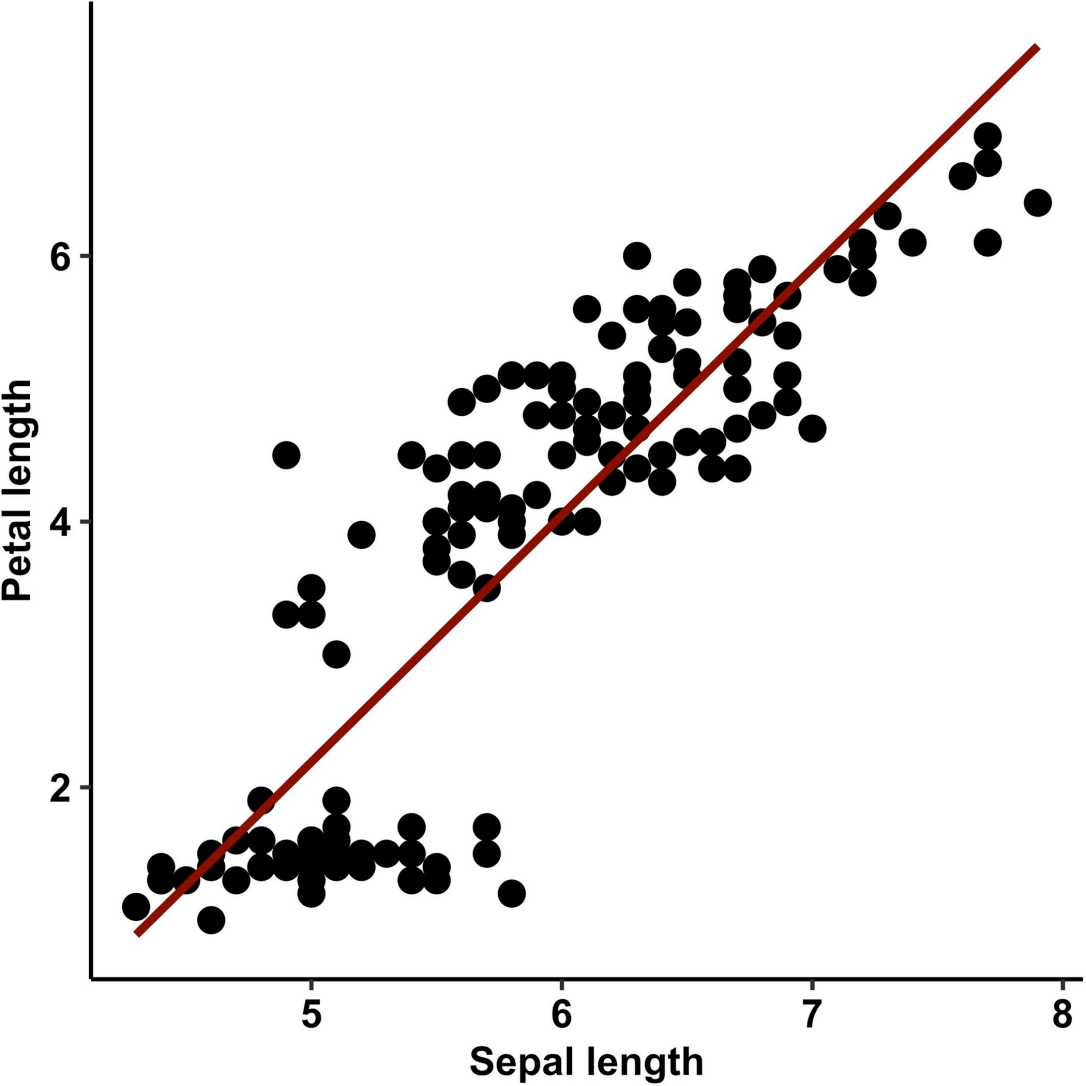
Example scatter plot. The red line represents the slope of a linear regression model with sepal length (of various Iris specimens) as outcome variable and petal length as predictor. This plot can be reproduced using the R code provided in the supplementary materials (S3 File).

Instead of or in addition to, reporting tables, researchers can choose to graphically illustrate their regression models. Perhaps the most well-known form of visualizing regression results is the scatter plot with a straight line representing the regression slope that is fitted to a point cloud. However, scatter plots are not well-suited to graphically represent effect sizes and can accommodate only a limited number of variables [14]. See Fig 1 for an example scatter plot of a linear regression model which was created using values from Edgar Anderson’s classic Iris data set which contains various measurements of three species of iris flowers [15, 16].

**Fig 2:**
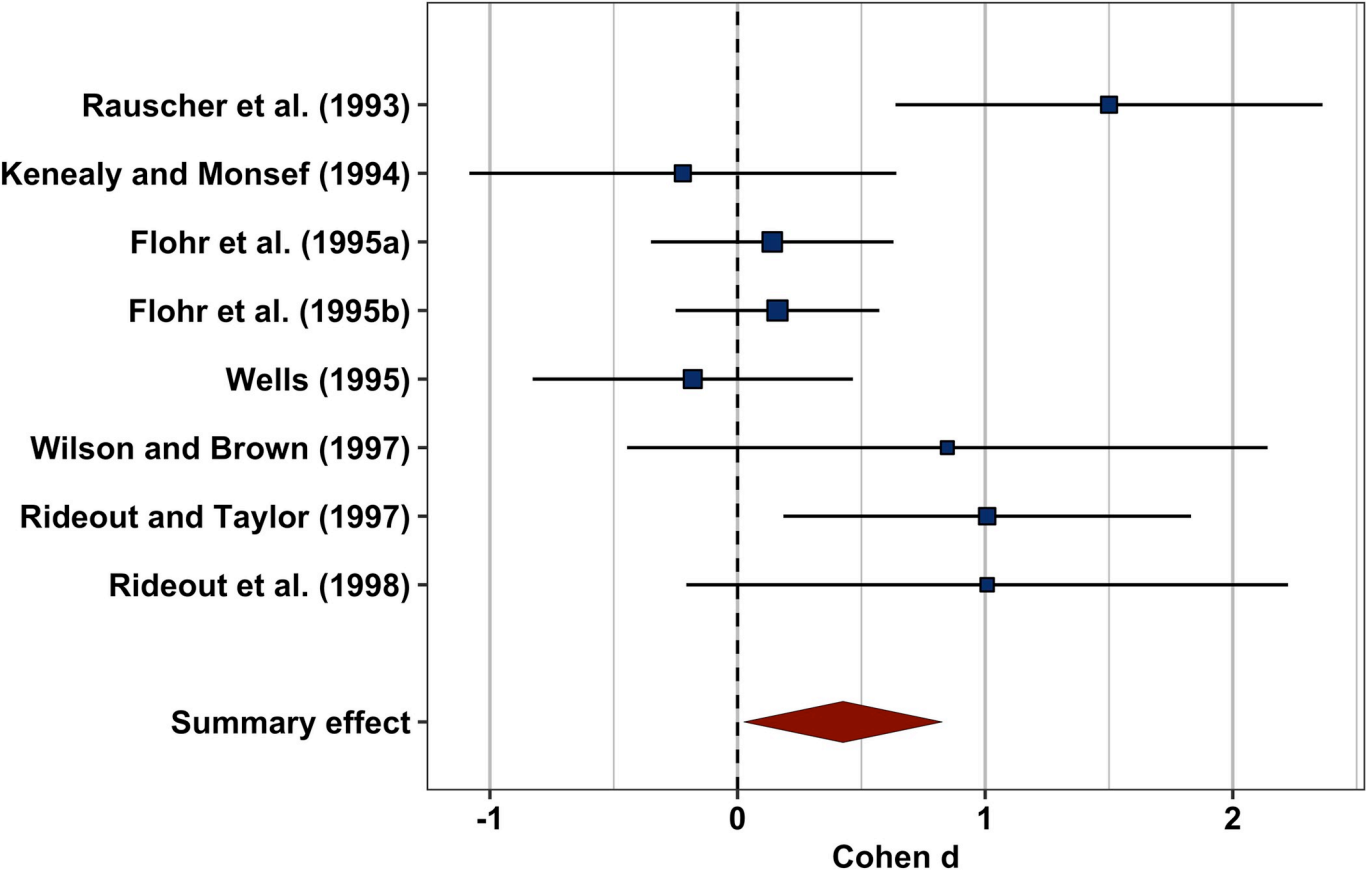
Forest plot for a subset of the Mozart effect meta-analysis included in the *metaviz* R package [18, 19]. This plot can be reproduced using the R code provided in the supplementary materials (S3 File).

An alternative approach to visualizing regression models was devised by modifying the forest plot. Initially developed in the context of research synthesis and meta-analysis, forest plots are typically used to summarize effect sizes of multiple studies (see Fig 2 for an example forest plot of a meta-analysis). The vertical dashed line represents a null effect. Each study is visualized as a square whose area corresponds to its precision (i.e., a function of its sample size). The squares’ positions on the x-axis indicates the studies’ respective effect sizes. Whiskers extending from the rectangles in both directions represent 95% confidence intervals (CIs). In addition, the diamond at the bottom of the graph indicates the weighted mean effect size and its CI [17]. This representation allows readers to quickly make sense of the results without having to comb through extensive tables.

**Fig 3:**
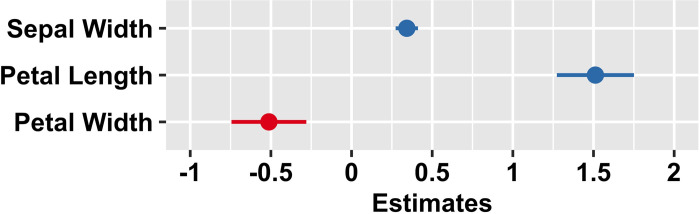
Forest plot of a multiple linear regression model of iris sepal length predicted by sepal width, petal length, and petal width. Blue or red circles represent standardized predictor estimates (i.e., beta weights). Horizontal bars represent the corresponding CIs. The color of the predictor indicators changes according to the estimate’s sign. This plot can be reproduced using the R code provided in the supplementary materials (S3 File).

Forest plots can be altered to represent multiple linear or logistic regression models. Contrary to the meta-analytical application, in a regression forest plot the horizontal entries do not represent study effect sizes but predictor point estimates and their associated CIs; see Fig 3 for an example regression forest plot that was created using the R package *sjPlot* [20] and the Iris data set.

A forest plot of a multiple regression model is typically used to visualize one model at a time, although there are implementations in R packages that allow plotting of more than one multiple regression model in one graph [e.g., 21].

Although some techniques of visualizing regression models do exist, such methods are seldom applied when reporting regression results. To illustrate this, we surveyed all 2022 and 2023 issues of *Psychotherapy and Psychosomatics* which ranks among the highest-impact psychology journals (Impact Factor 2023: 26.617). We found that of 17 studies that reported results of regression analyses [22–38], only six made use of at least one of these visualizations [22–27]. One of them applied a forest plot variant [22]. The neglect of visualization, despite its benefits, may be attributable to the fact that in many cases, larger numbers of regression models need presenting which can be difficult to achieve using the currently available techniques.

### Visualizing single-predictor regression models

Larger numbers of regression models can arise when the use of multiple regression is inappropriate, ill-advised, or unfeasible. For instance, multiple regression is not the method of choice in the presence of multicollinearity or when predictors are evaluated in isolation during the early stages of model selection. In these scenarios, single regressions are arguably more appropriate [6].

We encountered such a scenario in an unrelated project in which we aimed at predicting several educational outcomes by socioeconomic indicators. These predictors were strongly correlated with one another, precluding the use of multiple regression. To further complicate the analyses, multiple subcategories called *plausible values* (PVs) were available for each educational indicator, as is typical for large-scale educational assessments [39]. The recommended analysis strategy is to compute separate regression models for each PV and to subsequently pool the respective regression parameters using a procedure named Rubin’s rules [40, 41]. If multiple predictors are used, one can easily end up with hundreds of single regression models–but how can one present them in an accessible manner [42]?

While visualizing one or more multiple regression models at the same time is comparatively easily achieved by means of off-the-shelf solutions, this becomes more challenging if numerous models are involved. Plotting many models using scatter plots (i) consumes a disproportionate amount of space, (ii) results in an unwieldy number of visual regression slope representations that are difficult to compare, and (iii) offers little benefit over equally unwieldy tables. Unfortunately, viable alternatives are lacking. To our knowledge, none of the common statistical software environments currently offer solutions to this problem.

Thus, for reporting larger numbers of regression models, especially in the context of large-scale educational data, we developed a method that allows displaying the results in an intuitively interpretable visualization. Consequently, in the current article, we demonstrate the novel “beta-range forest plot”. It allows researchers to combine multiple single regression models in a single graph and thus allows quick visual screenings of potential trends in regression slopes without having to painstakingly sift large numbers of table entries.

Uncertainty and assumption violations are issues that must be considered in virtually every predictive model [e.g., 6]. Accurately representing the uncertainty of one’s findings is arguably one of a scientist’s foremost duties in reporting their results. Especially considering how scientific evidence is often picked up by popular media, the messaging should be as unambiguous as possible [43]. Naturally, this should also be the case for figures. Therefore, in addition to the beta-range forest plot, we present two alternative approaches to visualizing uncertainty in regression models by making use of bootstrapped distributions.

In all plots introduced in the current article, the proposed visualizations display standardized regression coefficients, because they are the foremost elements of a regression model when it comes to the interpretation of individual predictors [6]. In addition, the precision of these effect estimates is indicated using three different methods.

Readers are welcome to replicate the visualizations by running the accompanying code. It was written in the open-source statistical programming language R (R Core Team, 2022). In the main text, we only present snippets to improve readability. The full code can be accessed in the supplementary materials (S2 File). We recommend running the code from the script rather than from the main text.

## Methods

The protocol described in this peer-reviewed article is published on protocols.io, dx.doi.org/10.17504/protocols.io.5qpvorz4dv4o/v2, and is included for printing as S1 File with this article.

### Demonstration data

To provide a fully reproducible demonstration of the beta-range forest plot, we used data from a prior study [44]. These data are made up of two major components. The first part of the dataset is a subset from the large-scale educational assessment Trends in International Mathematics and Science Study (TIMSS). Initiated in 1995, TIMSS is an international study that allows comparisons of student achievement in mathematics and science across many nations. Because secondary publication of the original datasets is generally prohibited, we modified the data using several randomizing algorithms. Thus, while the overall structure is identical to the original data and the trends within the data are largely unchanged, all individual students’ data points and identifiers have been obscured.

The second part of the dataset consists of macroeconomic variables provided by the World Bank (https://data.worldbank.org). The matching of data points and the general data curation procedure is described in more detail in the corresponding article [44].

For the current study, we used 8th graders’ student achievement data from 38 countries, assessed in 2019 (*n* = 87,547) and a selection of five macroeconomic indicators: GDP per capita (GDP), Gender parity index (GPI), Gini index (Gini), Government education expenditure in secondary education (GeS) and in total (GeT). Student achievement variables were limited to five mathematics and five science domains (see Table 1); for each of these domains, five plausible values (PVs) were provided, which represent unbiased estimates of population characteristics (e.g., means and variance for groups). For instance, Algebra scores are indicated using five PVs for each student (e.g., “BSMALG01”, “BSMALG02”, and so forth). In addition, the TIMSS datasets contains school identifiers, as well as sampling weights, which allow for clustering as well as weighting within regression models. Henceforth, we will refer to this dataset as “TIMSS dataset”; see Table 1 for summary descriptives.

**Table 1:** Summary descriptive statistics of TIMSS dataset.

Variable	*M*	*Mdn*	*SD*	*IQR*
**Student achievement scores**				
BSMALG01 to 05	490.96 to 491.79	490.89 to 491.70	109.97 to 112.00	149.91 to 153.12
BSMDAT01 to 05	480.01 to 481.07	482.58 to 483.89	115.65 to 117.54	162.65 to 164.90
BSMGEO01 to 05	488.79 to 489.60	487.41 to 488.39	110.28 to 111.94	152.08 to 154.95
BSMMAT01 to 05	477.00 to 478.05	474.53 to 475.60	105.71 to 107.71	151.11 to 153.24
BSMNUM01 to 05	487.67 to 488.27	487.90 to 488.37	107.86 to 109.64	149.54 to 152.43
BSSBIO01 to 05	479.31 to 481.01	489.16 to 490.59	116.86 to 118.82	160.94 to 163.75
BSSCHE01 to 05	477.43 to 479.36	484.14 to 485.69	117.37 to 119.80	161.76 to 164.58
BSSEAR01 to 05	473.66 to 474.60	483.58 to 484.35	122.17 to 124.20	170.81 to 173.11
BSSPHY01 to 05	478.84 to 480.90	484.22 to 486.39	113.12 to 115.12	157.03 to 159.53
BSSSCI01 to 05	477.96 to 479.69	485.63 to 487.11	110.55 to 112.18	154.00 to 156.31
**Macroeconomic indicators**				
GDP	28910.56	23551.90	21805.43	35177.00
GeS	19.80	21.10	7.55	7.90
GeT	4.58	4.50	1.42	2.30
Gini	36.82	33.90	10.70	10.90
GPI	1.00	1.00	0.03	0.03

*Note*. Student achievement scores beginning with “BSM” represent the mathematics subscores algebra (ALG), data and probability (DAT), geography (GEO), mathematics overall (MAT), and number (NUM); scores beginning with “BSS” represent the science subscores biology (BIO), chemistry (CHE), earth science (EAR), physics (PHY), and science overall (SCI). Within domains, scores are structured in five PVs for each domain, indicated by 01 to 05.

To demonstrate the potential versatility of the visualizations presented here, we generated an additional, fictional dataset of decathlon results. Decathlon is a combination athletics discipline. Over the course of two days, each athlete competes in ten track and field events (100 meters race, long jump, shot-put, high jump, 400 meters race, 110 meters hurdles, discus, pole-vault, javelin, and 1500 meters race [45]). We simulated data for 10,000 fictional athletes. We aimed to predict the results of the respective decathlon events from seven different hematological indices that have been shown to be associated with sports performance (ferritin, haptoglobin, hematocrit, hemoglobin, iron, red blood cell count, and transferrin [46]).

Obviously, the data needed to be strongly correlated within participants across events. In addition, the blood markers were also required to exhibit a certain degree of association. To simulate such data, we sampled data from multivariate normal distributions and truncated the values to remain within realistic boundaries. Because data simulation is not the primary focus of this article, we encourage readers to refer to the protocol file in the supplementary materials where the process is described in detail (S1 File) as well as the more canonically formatted R code file (S2 File). The resulting dataset is summarized in Table 2. This dataset will henceforth be named “decathlon dataset”.

**Table 2:** Summary descriptive statistics of simulated decathlon dataset.

**Variable**	***M***	***Mdn***	***SD***	***IQR***
**Decathlon events**				
100 m	10.78	10.78	0.29	0.48
Long jump	7.43	7.43	0.44	0.73
Shot put	13.66	13.66	0.46	0.78
High jump	1.95	1.95	0.03	0.05
400 m	48.02	48.00	1.64	2.66
110 m hurdles	14.79	14.79	0.71	1.17
Discus throw	44.20	44.20	2.79	4.56
Pole vault	4.90	4.90	0.17	0.28
Javelin throw	57.17	57.13	3.52	5.84
1500 m	4.60	4.60	0.23	0.38
**Hematological indices**				
Ferritin	68.29	68.27	8.90	12.15
Haptoglobin	65.70	65.75	9.30	12.53
Hematocrit	46.78	46.81	2.68	3.78
Hemoglobin	15.92	15.92	1.08	1.52
Iron	122.37	122.37	10.46	14.44
Red blood cell count	5.24	5.24	0.53	0.72
Transferrin	320.77	321.48	41.41	55.46

Both datasets presented here are intended for demonstrational purposes and should be regarded as fictional.

### The beta-range forest plot

To demonstrate the application of the beta-range forest plot, we ran linear mixed-effects regression analyses predicting student achievement scores from country-level macroeconomic indicators. It is standard practice in the analysis of large-scale educational assessments, such as TIMSS, Programme for International Student Assessment (PISA), or the Progress in International Reading Literacy Study (PIRLS), to consider the clustering of scores within schools as a potential confounder [39]. Therefore, we included the school identifier as a random-effects term in all models. Using the sampling weights provided in the TIMSS dataset, we computed weighted least squares linear regressions for each of the five PVs making up each of the ten domains. Due to substantial multicollinearity, single-predictor regression models were applied. It is generally discouraged to solely interpret models based on individual PVs. Usually, the parameters from these models are incorporated using a statistical procedure known as Rubin’s rules [40]. Using this technique, the results of all five regression models for each PV are combined within domains, yielding pooled estimates, standard errors, and so forth. In all, this resulted in 250 regression models; see S1 Table for detailed model parameters.

These regressions were then combined into 50 pooled effect estimates. Considering the large number of regression models, presenting these results in an intuitive manner is quite challenging when using standard solutions. One could opt to only present the combined estimates. However, this would necessitate omitting the original regression models which were used to derive the pooled estimates. Here, we present a solution that can accommodate both the original models as well as the pooled estimates–the beta-range forest plot.

The process of creating such a plot begins by preparing the data. First, we need to create a table that contains all necessary model parameters; for a shortened version, see Table 3. The full table is included in spreadsheet form in the supplementary materials (S1 Table).

**Table 3:** Shortened table of model parameters.

Predictor	Outcome	β	*t*	*p*	LL^1^	UL^1^
GDP	BSMMAT01	0.25	137.32	< .001	0.25	0.26
GDP	BSMMAT02	0.25	137.89	< .001	0.25	0.26
GDP	BSMMAT03	0.25	137.35	< .001	0.25	0.26
**…**						
GeS	BSSPHY03	0.15	51.12	< .001	0.15	0.16
GeS	BSSPHY04	0.15	51.82	< .001	0.15	0.16
GeS	BSSPHY05	0.15	51.28	< .001	0.15	0.16

*Note*. This table contains only the first three and the final three rows of S1 Table) “LL” indicates the lower limit of the 95 percent CI of the point estimate, while “UL” indicates the upper limit.

Because our goal was to summarize all models as well as pooled estimates, we needed to further condense the data. Thus, we categorized the five PVs within each domain in a new variable called “domain” (e.g., “BSMMAT01“, “BSMMAT02”were categorized to “BSMMAT” and so forth).

Our aim was to summarize the five PVs to one visual indicator. To achieve this, we stored the smallest as well as the largest value among the five point estimates as separate variables. We repeat this process with the lowest value among the lower limits of the CIs and the largest values among the upper limits of the CIs. In addition, Rubin’s rules are applied to the individual regression models to attain pooled effect estimates for each domain. The technical details are described in the step-by-step protocol (S1 File) and the R code (S2 File).

By pooling effect sizes, as well as summarizing point estimates and corresponding CIs to value ranges, the number of rows in the regression parameter dataset was reduced from 250 to 50. Table 4 shows a shortened version of the summarized regression parameter dataset. For each domain, the summarized dataset contains pooled estimates, in addition to ranges representing the point estimates and CIs of all five PVs.

**Table 4:** Summarized regression parameter dataset, shortened.

Domain	Predictor	β (pooled)	LL estimate^1^	UL estimate^1^	LL CI^2^	UL CI^2^
Algebra	GDP	0.21	0.21	0.21	0.21	0.22
Algebra	Gini	-0.12	-0.12	-0.11	-0.13	-0.10
Algebra	GPI	0.01	0.00	0.01	0.00	0.02
**…**						
Science	GPI	-0.02	-0.03	-0.02	-0.03	-0.02
Science	GeT	-0.12	-0.12	-0.12	-0.13	-0.11
Science	GeS	0.17	0.16	0.17	0.16	0.18

*Note*. This table contains only the first three and the final three rows of S1 Table) “LL estimate” indicates the lower boundary of the point estimate range, “UL estimate” indicates the upper boundary. 2) “LL CI” indicates the lowest value among CIs within this category, “UL CI” indicates the highest value.

**Fig 4:**
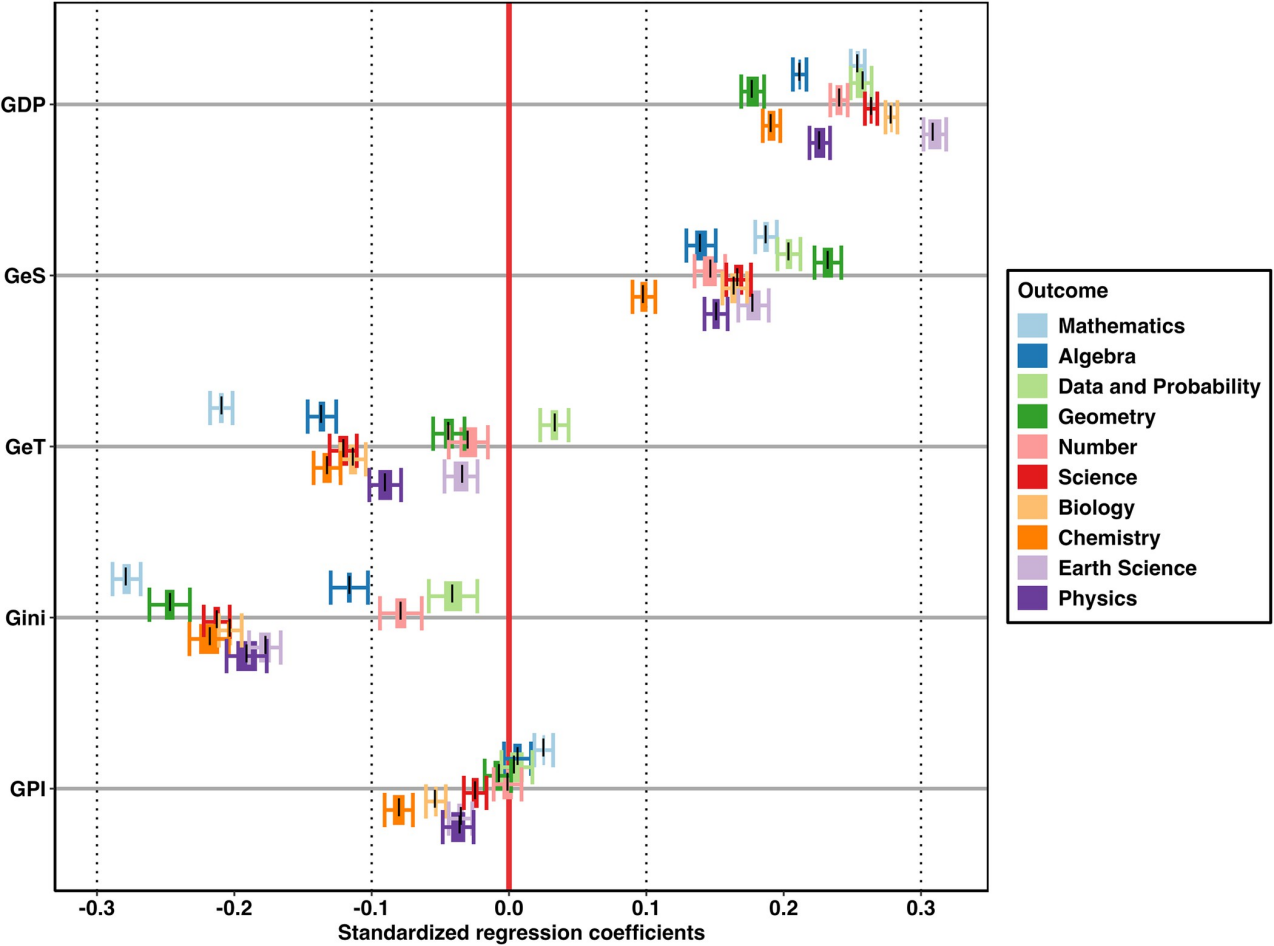
Beta-range forest plot for TIMSS data.

The dataset is now ready for plotting. For this purpose, we use the data visualization environment ggplot2 [47]. The beta-range forest plot that we created is displayed in Fig 4. Analogous to the forest plot of a multiple linear regression model in Fig 3, predictors are arranged along the y-axis. The x-axis corresponds to standardized regression coefficient effect sizes (also known as beta weights) which can assume absolute values between zero and one, with larger values indicating greater effect strengths; negative values indicate inverse relationships. A red vertical line is located at the null effect. Dotted vertical lines at the x-axis locations 0.1, 0.3, and 0.5 mark the well-established effect size interpretation thresholds for small, moderate, and large effects introduced by Cohen [48], though the 0.5 mark is not visible because the axis is cut off. Horizontal bars indicate the range of point estimates (i.e., representing the range between the smallest and the largest of the five effect estimates), while the horizontally extending whiskers indicate the outer limits of the most extreme corresponding CIs. If the whiskers of a given horizontal entry were to overlap with the red reference line (i.e., the null effect), at least one of the (five) individual predictors that comprise the respective entry failed to reach statistical significance. In addition, the black lines near the center of each indicator marks the location of the pooled effect size estimate computed using Rubin’s rules.

This visualization allows the viewer to quickly judge which macroeconomic indicators predict specific mathematics and science domains. In this modified data example, gross domestic product per capita and government spending on education have an overall larger impact on student achievement in mathematics and science compared to the Gender parity index. In terms of domains, earth science seems to be more substantially impacted by an increase in GDP compared to geometry.

Some of the CI ranges overlap with the line of no effect, indicating that these models failed to surpass the threshold of significance. However, in the current example, we had the advantage of having access to a large sample. Thus, most models are statistically significant. In such cases, considering sample size is critical. To this end, we added dotted lines indicating commonly used effect size benchmarks to facilitate interpretation.

Using the beta-range forest plot, it is possible to summarize a large amount of regression results that can be intuitively interpreted—in this case, 250 regression models. In addition, pooled regression coefficients were displayed in the same graphs. This cannot be achieved with any of the currently existing visualization methods.

This graph is a summary visualization that does not preserve all fine-grained detail within the data. Thus, it is not intended as a replacement for tables but as a complementary technique to illustrate the bigger picture of regression results. We recommend including the beta-range forest plot in the main text of a manuscript while reporting the detailed results in the supporting materials. While the plot can be readily applied in the analysis of large-scale educational assessment data, it is easily adapted to suit diverse analytical needs, including multivariate methods.

### The bootstrap ridgeline plot and the bootstrap violin plot

In the beta-range forest plot, the colored bars and the corresponding whiskers represent standardized regression estimates and CIs. However, one drawback of conventional table- or figure-based effect summaries is their limited potential to provide insights regarding skewed distributional characteristics. For instance, when scrutinizing a wider confidence interval, it appears that the center of the CI is the most probable location of the true effects. However, the whiskers do not inform us whether these effects are more likely to be located near the outer boundaries of the CI or more towards its middle. The CIs are rather narrow in the large TIMSS sample. In scientific practice, statistical power is often limited, and CIs tend to be larger. Consequently, the likelihood of true effect locations is more difficult to judge when solely relying on CIs. This is a well-known shortcoming of reporting CIs [e.g., 49]. Efforts have been made to improve the illustration of uncertainty, including density strips that display effect confidence as a function of opacity [50] or plotting a theoretical distribution of effect probability [51]. Below, we propose an alternative solution.

Instead of plotting theoretical effect locations, we ran bootstrap simulations for each effect estimate. Bootstrapping is a resampling method that involves creating multiple samples of the original dataset and subsequently computing the statistic of interest, standardized regression coefficients in our case. This results in a distribution of estimates that allows us to estimate the probability with which estimates may occur at a certain location [52]. Subsequently, we visualize this distribution in two different ways.

For demonstrating the bootstrapped plotting methods, we used the decathlon dataset which is composed of 10,000 fictional athletes who had competed in the ten track-and-field events that comprise a decathlon. We predicted performance in each of these events by blood markers. To keep this demonstration simple, we used single-predictor regression models. However, the proposed methods are also applicable to multivariate regression analyses. In all, ten events were predicted by seven blood indices, resulting in 70 regression models; the regression parameters of these models can be found in S2 Table.

Subsequently, bootstrap estimates were computed for the regressions. For each regression model, 500 samples with replacement were drawn. For each of these samples, standardized regression coefficients were calculated.

We thus generated 500 parameter estimates for each of the 250 regression models, resulting in 35,000 beta coefficients. Before being able to plot these coefficients, some data modifications were necessary (renaming of variables, reordering of factor levels, class conversions); these operations can be found in the protocol as well as in the R code in the supplementary materials (S1 and S2 Files). Table 5 contains a shortened version of the bootstrapped parameter estimate data set.

**Table 5:** Shortened version of the bootstrapped beta coefficients data set.

β	Predictor	Outcome
0.39	Ferritin	100 m
0.39	Ferritin	100 m
0.40	Ferritin	100 m
**…**		
0.19	Tansferrin	1500 m
0.20	Transferrin	1500 m
0.18	Transferrin	1500 m

*Note*. The actual dataset has 35,000 rows and can be reproduced using S1 and S2 Files.

The data is now ready to be plotted. Instead of simply displaying Wald or bootstrap CIs, we display the distributions of bootstrap beta coefficients as miniature density plots, a mode of visualization also known as ridgeline plot [53, 54].

**Fig 5:**
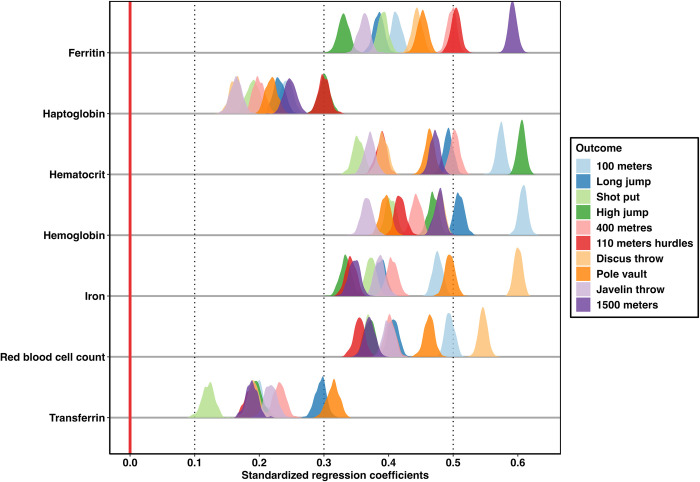
Bootstrap ridgeline plot, displaying miniature density plots of 500 parameter estimates for each decathlon regression model.

The bootstrap ridgeline plot we created is shown in Fig 5. Predictors and outcomes are arranged analogously to the beta-range forest plot. However, the range of possible values for beta coefficients is now displayed in the form of color-coded, miniature density plots which are arranged horizontally.

This method of visualizing regression has some obvious advantages. First, we have added bootstrap estimates to the visualization which can be more accurate and reliable than more traditional methods [52]. By displaying them as density plots, readers can easily judge the likelihood of effect estimates and their respective confidence. Additional indicators representing 95% confidence intervals could be added to each of the density plots. However, we chose not to overcrowd the plotting area for figure readability.

Our simulated decathlon example features a plethora of predictors and outcomes. This causes density plots to partially or fully overlap, making them hard to distinguish in some places. The bootstrap ridgeline plot may therefore be better suited to cases where fewer outcomes and predictors are involved. To address the issue of overlapping density plots, we developed an alternative representation of the distributions of bootstrapped beta coefficients by making use of the violin plot. To create this graph, the bootstrap dataset from before can be used without modifications.

**Fig 6:**
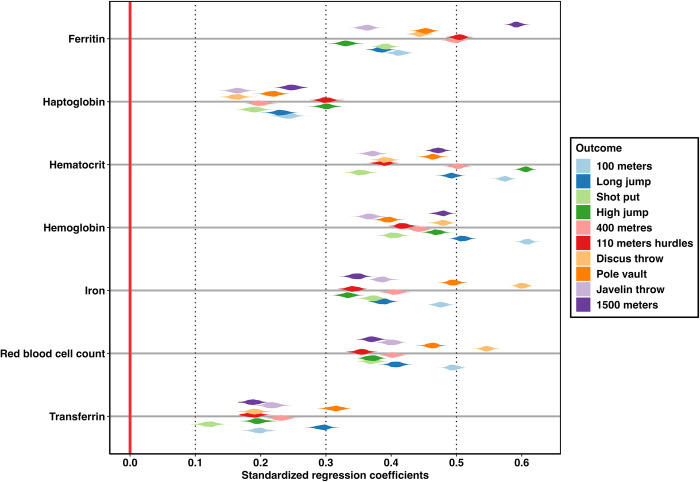
Bootstrap violin plot, displaying symmetrically mirrored density plots of 500 parameter estimates for each decathlon regression model.

The bootstrap violin plot we created is shown in Fig 6. In essence, this graph displays the same data as the bootstrap ridgeline plot, but in an arguably more discernable manner. Bootstrapped beta coefficients are displayed in the form of violin plots which are essentially graphical representations of a given variable’s density function, mirrored along the symmetry axis which can resemble the shape of a violin [55]. With the use of this representation, the issue of overlapping density plots is resolved. However, owing to the narrower indicators, distribution shapes are represented in less detail compared to the bootstrap ridgeline plot.

## Discussion

The primary objective of this article was to introduce novel methods for presenting regression results, particularly when dealing with a large number of regression models. Current methods of reporting regression typically fall short in critical respects. They either lack intuitive interpretability, while offering little visual stimulus to the reader (e.g., tables), or they contain very limited information (e.g., scatter plots). Here, we used established graphical and statistical elements in innovative ways to create new, information-dense and arguably aesthetically appealing visualizations. All three graphs are variations of the forest plot, modified to indicate point estimates as well as the dispersion of parameter estimates in different ways, presented with increasing complexity across the present article.

The beta-range forest plot is particularly valuable when numerous single-regression models with non-independent samples must be reported. Especially when analyzing large-scale educational assessment data, this graph can be a beneficial addition. Owing to the use of non-parametric and robust bootstrapping techniques, the bootstrap ridgeline and violin plots can be especially useful in cases where the underlying population distributions are unknown or non-normal. In all visualizations presented here, we processed multiple single-regression models. With a few modifications, both plots could also accommodate logistic and/or multiple regression models.

Future researchers may wish to gauge the interpretability of these figures by conducting a survey among researchers. Such a survey could help to determine how effectively the visualizations are conveying the relevant information to their intended audience. The survey can be designed to include questions on various aspects of the figures such as the appropriateness of the visual encoding, the ease of understanding the message conveyed, and the overall effectiveness of the figure in communicating the intended information.

In summary, we propose three alternative methods of visualizing regression models. They enable researchers to visualize large numbers of regression models in single graphs. We demonstrate this by plotting 350 regression models of simulated data using only one figure, in three different ways. These novel methods offer information-dense, yet intuitive and aesthetically compelling ways to present regression results.

## Supporting information

S1 File Step-by-step protocol describing the workflow for creating the introduced visualizations and simulating the demonstration data. (PDF)

S2 File R code for creating the introduced visualizations and simulating the demonstration data. (R)

S3 File R code for reproducing the figures presented in the introduction section. (R)

S1 Table Model parameters for the 250 mixed-effects linear regression models based on the TIMSS dataset. (XLSX)

S2 Table Model parameters for the 70 mixed-effects linear regression models based on the decathlon dataset. (XLSX)
