# Supplementary material for: Say farewell to bland regression reporting: Three forest plot variations for visualizing linear models
Source: PLoS One. 2024 Feb 2;19(2):e0297033. doi: 10.1371/journal.pone.0297033 (PMC10836698; doi:10.1371/journal.pone.0297033)
Supplement: S1 File — (PDF) [file pone.0297033.s001.pdf]

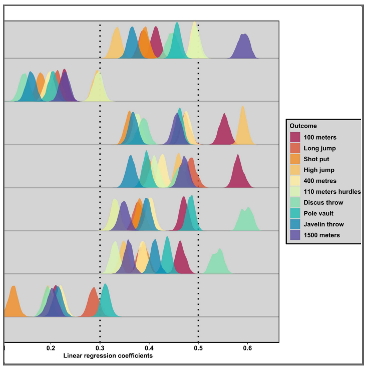

CZCIX2UE

Protocol Info: Jonathan Fries, Sandra Oberleiter, Jakob Pietschnig . Workflow for beta-range forest plots, bootstrap ridgeline plots, and bootstrap violin plots. **protocols.io** https://protocols.io/view/workflow-for-beta-range-forest-plots-bootstrap-rid-czcix2ueVersion created by Jonathan Fries

Created: Aug 30, 2023

Last Modified: Sep 04, 2023

PROTOCOL integer ID: 87146

Workflow for beta-range forest plots, bootstrap ridgeline plots, and bootstrap violin plots V.(czcix2ue)

Jonathan Fries[1], Sandra Oberleiter[1], Jakob Pietschnig[1]

[1]Department of Developmental and Educational Psychology, Faculty of Psychology, University of Vienna

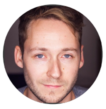

Jonathan Fries
Universität Wien

ABSTRACT

Regression is a widely used statistical method in various research areas, such as educational psychology, and it is common to display regression coefficients in tables. Although tables contain dense information, they can be difficult to read and interpret when they are extensive. To address this issue, we present three innovative visualizations that enable researchers to present a large number of regression models in a single plot. We demonstrate how to transform simulated data and plot the results, which produce visually appealing representations of regression results that are both efficient and intuitive. These visualizations can be applied for screening models in the selection stage or reporting results in research papers. Our method is reproducible using the provided code and can be implemented using free and open-source software routines in R.

GUIDELINES

Get R:

The statistical programming environment can be downloaded free-of-charge at https://cran.r-project.org.

Recommendations and links

1 We recommend running the code in this protocol from the text file ("regression_plots.R"). All necessary data and code files can be downloaded at this repository: Open Science Framework repository

Install and load packages

2 The following workflow requires the statistical programming environment R (at least version 4.2.2). Before running any command prompts, some packages need to be installed. To this end,

we create a vector that contains the respective package names and run a for loop that only installs packages that are not already installed.

```
required_packages <- c("boot", "broom", "car", "data.table", "faux",
"ggplot2", "ggridges", "lme4", "lmerTest","JWileymisc", "MASS",
"psych", "RColorBrewer", "readxl")

for(i in required_packages){
 package_temp <- i
 if(!package_temp %in% installed.packages()) {
    install.packages(package_temp)
 }
}
```

Next, we load the required packages.

```
library(boot)
library(broom)
library(car)
library(data.table)
library(faux)
library(ggplot2)
library(ggridges)
library(JWileymisc)
library(lme4)
library(lmerTest)
library(MASS)
library(psych)
library(RColorBrewer)
library(readxl)
```

Importing modified large-scale educational assessment data

3 For demonstrating the first data visualization technique (beta-range forest plot), we modified data from the 2019 TIMSS (Trends in Mathematics and Science Study) assessment of 8th graders from 39 countries (*n* = 87,547). These data were linked to macroeconomic indicators provided by the world bank (https://data.worldbank.org). The data were modified because publication of the original datasets is prohibited. The modified dataset ("timss_modified.xlsx") can be downloaded at:
Open Science Framework repository

4 We import the dataset. Please select the file "timss_modified.xlsx".

```
timss <-
 read_xlsx(file.choose())
```

4.1 Now the dataset is converted to long format. We use the melt function from the reshape2 package.

```
timss_long <-
 as.data.table(
  reshape2::melt(timss,
   id.vars = c(
    "Country",
    "IDSCHOOL",
    "IDSTUD",
    "ITSEX",
    "TOTWGT",
    "TOTWGT_normalized",
    "gdp",
    "gini",
    "gpi",
    "get",
    "ges"),
   variable.name = "outcome",
   value.name = "result"))

# Add macroeconomic indicators as grouping variable #

timss_long <-
 as.data.table(
  reshape2::melt(timss_long,
   id.vars = c(
    "Country",
    "IDSCHOOL",
    "IDSTUD",
    "ITSEX",
    "TOTWGT",
    "TOTWGT_normalized",
    "outcome",
    "result"),
   variable.name = "predictor",
   value.name = "pred_value"))
```

Regression for modified TIMSS data

5 Now, we run linear regressions in batch for each unique combination of outcome and predictor. In the same step, we extract selected parameters into a data.table. CAUTION: May incur substantial computation time due to the mixed-effects models.

```
models_timss <-
 timss_long[, {
    model <- lmer(scale(result) ~ scale(pred_value) + (1|IDSCHOOL),
REML = FALSE, weights = TOTWGT_normalized)
     summary_model <- summary(model)
    .(
     outcome = outcome[1],
     predictor = predictor[1],
     estimate = summary_model$coefficients[2, 1],
     se = summary_model$coefficients[2, 2],
     statistic = summary_model$coefficients[2, 4],
     p = summary_model$coefficients[2, 5],
     ll = summary_model$coefficients[2, 1] - 1.96 *
summary_model$coefficients[2, 2],
     ul = summary_model$coefficients[2, 1] + 1.96 *
summary_model$coefficients[2, 2])
 },
by = .(outcome, predictor)]
```

Data modification of TIMSS regression dataset

6 Now, we perform some data modifications on the resulting regression dataset.

```
# Create a factor that categorizes PVs into domains #

models_timss$domain <-
 as.factor(
  gsub(
   "([0-9]+[0-9])",
   "",
   models_timss$outcome))

# Convert summarized regression parameter dataset to data.table #

models_timss <- as.data.table(models_timss)

# Class conversions #

if(class(models_timss$predictor) != "factor"){
 models_timss$predictor <- as.factor(models_timss$predictor)
}

if(class(models_timss$outcome) != "factor"){
 models_timss$outcome <- as.factor(models_timss$outcome)
}

if(class(models_timss$domain) != "factor"){
 models_timss$domain <- as.factor(models_timss$domain)
}
```

Compute estimate ranges, CI ranges, and pooled effect estim...

7 The next step is arguably the most important for plotting the data. Here, we compute the parameters that are subsequently displayed in the plot.

7.1 We create an empty container dataframe to append pooled effect estimates to (calculated in loop below).

```
models_timss_pooled <-
 setDT(
  data.frame(
   domain_name = 0,
   predictor_name = 0,
   estimate_pooled = 0,
   se_pooled = 0,
   wald_pooled = 0,
   p_pooled = 0,
   ll_pooled = 0,
   ul_pooled = 0,
   ll_estimate = 0,
   ul_estimate = 0,
   ll_ci = 0,
   ul_ci = 0))
```

7.2 Next, lower and upper limits of point estimate ranges as well as CI ranges are stored in a dataframe. In addition, we calculate pooled effect estimates using the procedure laid out by Rubin (1987).

```
for(j in levels(models_timss$domain)){
 domain_name <- j
 dat_temp.1 <- subset(models_timss, domain %in% domain_name)
 for(i in levels(dat_temp.1$predictor)){
  predictor_name <- i
  dat_temp.2 <-
   subset(
    dat_temp.1,
    predictor %in% predictor_name)
  m <- nrow(dat_temp.2)
  estimate_pooled <- sum(dat_temp.2$estimate)/m
  within_imputation_variance <- sum(dat_temp.2$se^2)/m
  between_imputation_variance <- var(dat_temp.2$estimate)
  total_imputation_variance <- within_imputation_variance +
between_imputation_variance + (between_imputation_variance/m)
  se_pooled <-
   sqrt(total_imputation_variance)
  wald_pooled <- estimate_pooled/se_pooled
  lambda <- between_imputation_variance +
(between_imputation_variance/m)/total_imputation_variance
  df_old <- (m-1)/(lambda^2)
  p_pooled <- 2*pt(abs(wald_pooled), df_old, lower.tail=FALSE)
  critical_t_value_pooled <- qt(p=.05/2, df=df_old,
lower.tail=FALSE)
  ll_pooled <- estimate_pooled -
(critical_t_value_pooled*se_pooled)
  ul_pooled <- estimate_pooled +
(critical_t_value_pooled*se_pooled)
  ll_estimate <- min(dat_temp.2$estimate)
```

```
  ul_estimate <- max(dat_temp.2$estimate)
  ll_ci <-  min(dat_temp.2$ll)
  ul_ci <- max(dat_temp.2$ul)
  regression_temp_table <-
   data.frame(
    domain_name,
    predictor_name,
    estimate_pooled,
    se_pooled,
    wald_pooled,
    p_pooled,
    ll_pooled,
    ul_pooled,
    ll_estimate,
    ul_estimate,
    ll_ci,
    ul_ci)
  models_timss_pooled <- rbind(models_timss_pooled,
regression_temp_table)
 }
 rm(domain_name, dat_temp.1, predictor_name, dat_temp.2, m,
estimate_pooled,
within_imputation_variance,between_imputation_variance,
total_imputation_variance, se_pooled, wald_pooled, lambda,
df_old, p_pooled, critical_t_value_pooled, ll_pooled, ul_pooled,
ll_estimate, ul_estimate, ll_ci, ul_ci, regression_temp_table)
}
```

7.3 Some formatting operations need to be performed.

```
# Remove empty first row of pooled estimate data set #

models_timss_pooled <- models_timss_pooled[-1,]

names(models_timss_pooled)[names(models_timss_pooled) ==
'predictor_name'] <- 'predictor'

names(models_timss_pooled)[names(models_timss_pooled) ==
'domain_name'] <- 'domain'

# Class conversions for pooled estimate table #

models_timss_pooled$domain <-
as.factor(models_timss_pooled$domain)

models_timss_pooled$predictor <-
as.factor(models_timss_pooled$predictor)
```

7.4 Now, we recode predictor and domain variables for nicer display in the plot. Then, the

dataframe is reordered.

```
models_timss_pooled$predictor <-
    car::recode(
        models_timss_pooled$predictor,
        "
            'gdp' = 'GDP';
            'ges' = 'GeS';
            'get' = 'GeT';
            'gini' = 'Gini';
            'gpi' = 'GPI' ")

models_timss_pooled$domain <-
 factor(
  car::recode(models_timss_pooled$domain,
             "
           'BSMMAT' = 'Mathematics';
              'BSMALG' = 'Algebra';
              'BSMDAT' = 'Data and Probability';
              'BSMGEO' = 'Geometry';
              'BSMNUM' = 'Number';
              'BSSBIO' = 'Biology';
              'BSSCHE' = 'Chemistry';
              'BSSEAR' = 'Earth Science';
              'BSSPHY' = 'Physics';
              'BSSSCI' = 'Science' "),
  levels = c(
   "Mathematics",
   "Algebra",
   "Data and Probability",
   "Geometry",
   "Number",
   "Science",
   "Biology",
   "Chemistry",
   "Earth Science",
   "Physics"))

# Reorder dataframe by Outcome #

models_timss_pooled <- setorder(models_timss_pooled, domain)
```

7.5 Some final formatting operations are necessary. We create factor variables with reverse-ordered levels. This is necessary because the coordinate system of the plots is flipped. Thus, factor levels also need to be "flipped".

```
models_timss_pooled$predictor_rev <-
    factor(
     models_timss_pooled$predictor,
     levels = rev(levels(models_timss_pooled$predictor)))

models_timss_pooled$domain_rev <-
    factor(
     models_timss_pooled$domain,
     levels = rev(levels(models_timss_pooled$domain)))
```

Beta-range forest plot

8 The data is now ready for plotting; we use the graphics environment ggplot2.

```
beta_range_plot <-
    ggplot(data = models_timss_pooled, aes(x = predictor_rev, color
= domain_rev)) +
    geom_hline(yintercept = 0, lty = 1, linewidth = 1.5, color =
"#E73134") +
    geom_hline(yintercept = c(-0.3, -0.1, 0.1, 0.3), lty = "dotted",
linewidth = 0.6, color = "black") +
    geom_linerange(
     aes(ymin = ll_estimate, ymax = ul_estimate),
     linewidth = 8,
     position = position_dodge(width = 0.5)) +
    geom_errorbar(
     aes(ymin = ll_ci, ymax = ul_ci),
     linetype = "solid",
     width = 2,
     linewidth = 1,
     position = position_dodge(width=0.5)) +
    geom_text(
     aes(y = estimate_pooled, group = domain_rev, label = "|"),
     color = "black",
     position = position_dodge(width = 0.5),
     vjust = 0.35,
  hjust = 0.5,
     size = 5,
     show.legend = FALSE) +
    coord_flip() +
    labs(
     y = "Standardized regression coefficients",
     x = "",
    title = "") +
    theme_classic() +
 theme(
  legend.background = element_rect(fill = "white", colour = "black",
linewidth = 0.8),
```

```
  panel.grid.major.y = element_line(linewidth = 1, linetype =
"solid", color = "darkgrey"),
  axis.title.y = element_blank(),
  panel.border = element_rect(colour = "black", fill = NA, linewidth
= 1),
  panel.background = element_rect(fill = 'white'),
  legend.position = "right",
  axis.text.y = text_settings,
        axis.text.x = text_settings,
        axis.title.x = text_settings,
        legend.text = text_settings,
  legend.title = text_settings) +
    scale_color_manual(
        values =
brewer.pal(length(levels(models_timss_pooled$domain)), name =
"Paired"),
        name = "Outcome",
        breaks = levels(models_timss_pooled$domain)) +
    scale_y_continuous(breaks = seq(-0.4, 0.4, 0.1))
```

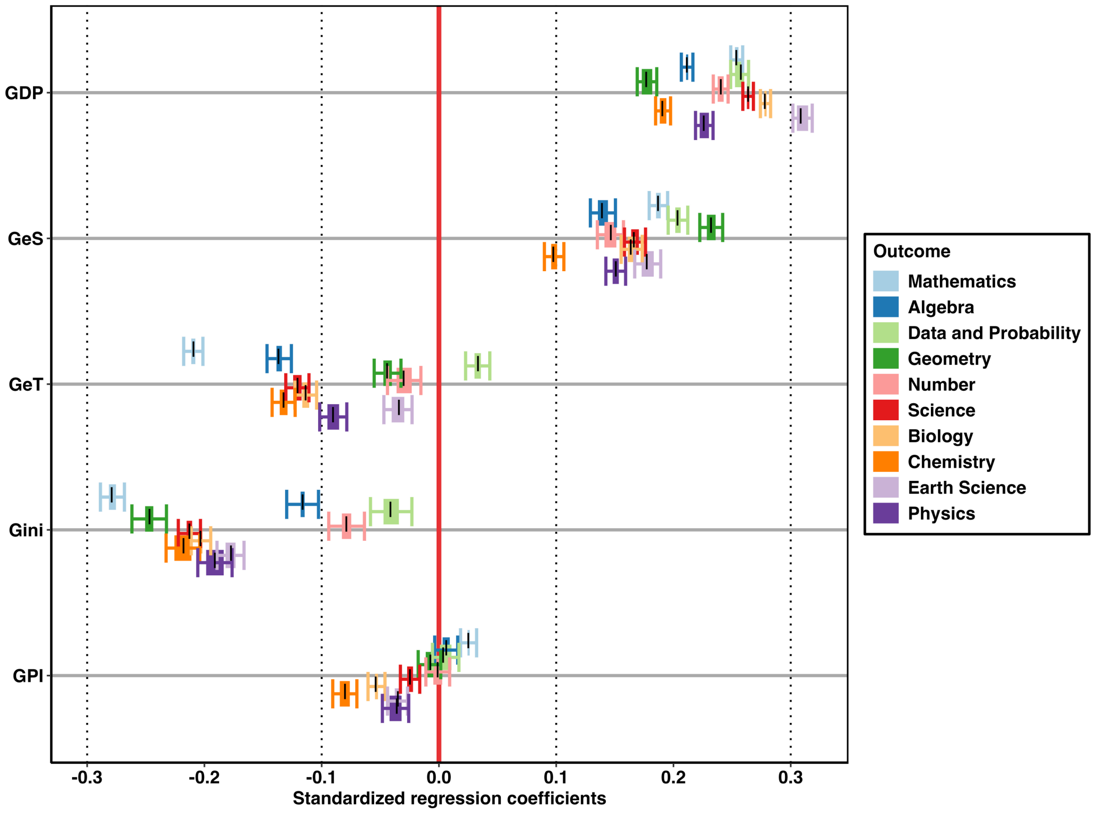

Predictors are arranged along the y-axis. The x-axis corresponds to standardized regression coefficient effect sizes (also known as beta weights) which can assume absolute values between zero and one, with larger values indicating greater effect strengths; negative values indicate inverse relationships. A red vertical line is located at the null effect. Horizontal bars indicate the range of point estimates (i.e., representing the range between the smallest and the largest of the five effect estimates), while the horizontally extending whiskers indicate the outer limits of the most extreme corresponding CIs. If the whiskers of a given horizontal entry

were to overlap with the red reference line (i.e., the null effect), at least one of the (five) individual predictors that comprise the respective entry failed to reach statistical significance. In addition, the black lines near the center of each indicator marks the location of the pooled effect size estimate computed using Rubin's rules.

Data simulation of decathlon results

9 To demonstrate the bootstrap-based data visualization techniques bootstrap ridgeline plot and bootstrap violin plot, we use simulated data.

9.1 A custom helper function for the data simulation process is defined beforehand.

```
# Function for truncating decathlon results to stay within
reasonable boundaries #

truncate_results <- function(x, ll = -Inf, ul = Inf, runif.min =
0.1, runif.max = 0.5) {
  temp_1 <-
   ifelse(
     x < ll,
     ll + runif(length(x), min=runif.min, max=runif.max),
     x)
  temp_2 <-
   ifelse(
     temp_1 > ul,
     ul - runif(length(x), min=runif.min, max=runif.max),
     temp_1)
  return(temp_2)
 }
```

9.2 A random seed is then defined; this step ensures that the results are exactly replicable. If replicability is of no concern, this command may be removed.

```
set.seed(239)
```

9.3 We create a dataframe that contains names and distribution parameters for the variables that we intend to simulate. Here, we chose to demonstrate our method using a fictional dataset of decathlon results. Decathlon is a combination athletics discipline. Over the course of two days, each athlete competes in ten track and field events (100 meters race, long jump, shot-put, high jump, 400 meters race, 110 meters hurdles, discus, pole-vault, javelin, and 1500 meters race).

```
decathlon_pars <-
 data.frame(
  discipline =
   factor(
    c(
     "hundred_m",
     "long_jump",
     "shot_put",
     "high_jump",
     "four_h_metres",
     "one_ten_meters_hurdles",
     "discus_throw",
     "pole_vault",
     "javelin_throw",
     "one_five_k_meters")
   ),
  ll = c(10.23, 6.62, 12.81, 1.88, 45.02, 13.46, 39.02, 4.61,
50.64, 4.17),
  ul = c(11.32, 8.25, 14.52, 2.01, 51.02, 16.11, 49.34, 5.20,
63.63, 5.03),
  mean = c(10.78, 7.42, 13.66, 1.95, 48.00, 14.78, 44.17, 4.90,
57.13, 4.60),
  sd = c(0.36, 0.54, 0.57, 0.04, 1.98, 0.87, 3.41, 0.21,  4.29,
0.28)
 )
```

9.4 The simulated decathlon results are expected to be intercorrelated. Thus, we define a matrix of correlations between the variables that we intend to simulate and convert it to a covariance matrix.

```
cov_mat <-
 JWileymisc::cor2cov(
  V = rbind(
    c(1,  0.5,  0.3,  0.6, 0.6,  0.5, 0.6, 0.5, 0.4, 0.7),
    c(0.5, 1,  0.3, 0.6, 0.5, 0.5, 0.4, 0.6, 0.4, 0.5),
    c(0.3, 0.3, 1,  0.4, 0.4, 0.5, 0.7, 0.3, 0.6, 0.4),
    c(0.6, 0.6,  0.4, 1,  0.4, 0.5, 0.3, 0.6, 0.4, 0.5),
    c(0.6, 0.5, 0.4, 0.4, 1,  0.7, 0.5, 0.6, 0.5, 0.7),
    c(0.5, 0.5, 0.5, 0.5, 0.7, 1,  0.4, 0.5, 0.4, 0.7),
    c(0.6, 0.4, 0.7, 0.3, 0.5, 0.4, 1,  0.5, 0.7, 0.4),
    c(0.5, 0.6, 0.3, 0.6, 0.6, 0.5, 0.5, 1,  0.7, 0.5),
    c(0.4, 0.4, 0.6, 0.4, 0.5, 0.4, 0.7, 0.7, 1,  0.4),
    c(0.7, 0.5, 0.4, 0.5, 0.7, 0.7, 0.4, 0.5, 0.4, 1)),
sigma = decathlon_pars$sd)
```

9.5 Using this covariance matrix, we now generate correlated decathlon results for 10,000 fictional athletes.

```
# Generate correlated decathlon results for 10,000 athletes #

decathlon_results <-
 setDT(
  as.data.frame(
   mvrnorm(
    n=10000,
    mu=decathlon_pars$mean,
    Sigma= cov_mat)))

# Rename variables #

colnames(decathlon_results) <-
 c(
  "hundred_m_01",
  "long_jump_01",
  "shot_put_01",
  "high_jump_01",
  "four_h_metres_01",
  "one_ten_meters_hurdles_01",
  "discus_throw_01",
  "pole_vault_01",
  "javelin_throw_01",
  "one_five_k_meters_01" )
```

9.6 Some of the generated values are outside the range of human physical capability. To address this, we truncate the results to stay within reasonable boundaries. The custom helper function we defined above is applied within a for loop.

```
for (i in decathlon_pars$discipline){
 disc_name <- paste0(i, "_01")
 pars_temp <-
  subset(
      decathlon_pars,
      discipline %in% paste0(i))
 decathlon_results[[disc_name]] <- truncate_results(
  decathlon_results[[disc_name]],
  ll = pars_temp$ll,
  ul = pars_temp$ul,
  runif.min = jitter(pars_temp$sd*0.2, amount=pars_temp$sd*0.1),
  runif.max = jitter(pars_temp$sd*0.4, amount=pars_temp$sd*0.1))
}
```

9.7 Simulation of athletics results is now completed. Next, some predictors are needed. We chose to use fictional data of seven hematological indices (ferritin, haptoglobin, hematocrit, hemoglobin, iron, red blood cell count, and transferrin) that have been demonstrated to be associated with sports performance. For each hematological index, we simulate normally distributed data that exhibit pre-defined correlations with some of the decathlon events.

```
decathlon_results$hemoglobin <-
 rnorm_pre(
  subset(
   decathlon_results,
   select=c(
    hundred_m_01,
    long_jump_01,
    shot_put_01)),
   mu = 15.9,
   sd = 1.1,
   r = c(0.6, 0.5, 0.4),
   empirical = FALSE)

decathlon_results$rb_cell <-
 rnorm_pre(
  subset(
   decathlon_results,
   select=c(
    discus_throw_01,
    pole_vault_01,
    javelin_throw_01,
    hemoglobin)),
   mu = 5.24,
   sd = 0.52,
   r = c(0.55, 0.45, 0.4, 0.6),
   empirical = FALSE)

decathlon_results$iron <-
 rnorm_pre(
  subset(
   decathlon_results,
   select=c(
    discus_throw_01,
    pole_vault_01,
    javelin_throw_01,
    rb_cell)),
   mu = 122.5,
   sd = 10.5,
   r = c(0.6, 0.5, 0.4, 0.7),
   empirical = FALSE)

decathlon_results$hematocrit <-
 rnorm_pre(
  subset(
   decathlon_results,
   select=c(
    high_jump_01,
    four_h_metres_01,
    one_ten_meters_hurdles_01,
```

```
     hemoglobin)),
    mu = 46.8,
    sd = 2.7,
    r = c(0.6, 0.5, 0.4, 0.7),
    empirical = FALSE)

decathlon_results$ferritin <-
 rnorm_pre(
  subset(
   decathlon_results,
   select=c(
    one_five_k_meters_01,
    one_ten_meters_hurdles_01,
    hundred_m_01,
    iron)),
   mu = 68.3,
   sd = 8.9,
   r = c(0.6, 0.5, 0.4, 0.7),
   empirical = FALSE)

decathlon_results$haptoglobin <-
 rnorm_pre(
  subset(
   decathlon_results,
   select=c(
    high_jump_01,
    four_h_metres_01,
    one_ten_meters_hurdles_01,
    hemoglobin)),
   mu = 65.7,
   sd = 9.3,
   r = c(0.3, 0.2, 0.3, 0.25),
   empirical = TRUE)

decathlon_results$transferrin <-
 rnorm_pre(
  subset(
   decathlon_results,
   select=c(
    long_jump_01,
    high_jump_01,
    pole_vault_01,
    hemoglobin)),
   mu = 321,
   sd = 40.9,
   r = c(0.3, 0.2, 0.3, 0.25),
   empirical = FALSE)
```

9.8 Data simulation is complete. To tidy up the data, we re-order the dataframe columns.

```
decathlon_results <-
 decathlon_results[,
  c(
   "hundred_m",
   "long_jump",
   "shot_put",
   "high_jump",
   "four_h_metres",
   "one_ten_meters_hurdles",
   "discus_throw",
   "pole_vault",
   "javelin_throw",
   "one_five_k_meters",
   "ferritin",
   "haptoglobin",
   "hematocrit",
   "hemoglobin",
   "iron",
   "rb_cell",
   "transferrin")]
```

Regression for simulated decathlon data

10 Next, we intend to predict decathlon outcomes from hematological indices. We model these relationships using a single-predictor linear regression framework.

10.1 The dataset needs to be converted to long format. The conversion is carried out using the melt command from the reshape2 package.

```
decathlon_results_long <-
 as.data.table(
  reshape2::melt(
   decathlon_results,
   id.vars = c(
    "ferritin",
    "haptoglobin",
    "hematocrit",
    "hemoglobin",
    "iron",
    "rb_cell",
    "transferrin"),
   variable.name = "outcome",
   value.name = "result"))

decathlon_results_long <-
 as.data.table(
  reshape2::melt(
   decathlon_results_long,
   id.vars = c(
    "outcome", "result"),
   measure.vars = c(
    "ferritin",
    "haptoglobin",
    "hematocrit",
    "hemoglobin",
    "iron",
    "rb_cell",
    "transferrin"),
   variable.name = "predictor",
   value.name = "pred_value"))
```

10.2 Now, we run regressions for each unique combination of outcome and predictor in batch and extract parameters using data.table, resulting in 70 regression models stored in a separate dataframe.

```
models_decathlon <-
 decathlon_results_long[,
  {
     model_summary <- summary(lm(scale(result) ~
scale(pred_value)))
    .(
     outcome = outcome[1],
     predictor = predictor[1],
     estimate = tidy(model_summary)$estimate[2],
     se = tidy(model_summary)$std.error[2],
     statistic = tidy(model_summary)$statistic[2],
     p = tidy(model_summary)$p.value[2],
     eta.sq = model_summary$r.squared[1],
     ll = tidy(model_summary)$estimate[2] - 1.96 *
tidy(model_summary)$std.error[2],
     ul = tidy(model_summary)$estimate[2] + 1.96 *
tidy(model_summary)$std.error[2])
  },
  by = .(outcome, predictor)]
```

Computing bootstrapped regression parameter estimates

11 The purpose of the demonstrated plots is the representation of the uncertainty associated with regression effect sizes. We achieve this by computing bootstrapped point estimates and subsequently plotting the resulting distributions.

11.1 First, we set a random seed.

```
set.seed(626)
```

11.2 Next, we define a helper function for bootstrapping.

```
boot_function <- function(formula, data, indices) {
   d <- data[indices,]
   fit <- lm(formula, data=d)
   return(tidy(fit)$estimate[2])
 }
```

11.3 We create a container dataframe for the botstrapped estimates which we add in the next step.

```
boot_df <-
 as.data.table(
  data.frame(
   V1 = 0,
   predictor = 0,
   outcome = 0))
```

11.4 Now we compute bootstrap parameter estimates for all 70 regression models from above (models_decathlon). CAUTION: This computation is fairly lengthy.

```
for(i in 1:nrow(models_decathlon)){
 orig_model_temp <- models_decathlon[i,]
 predictor_temp <- orig_model_temp$predictor
 outcome_temp <- orig_model_temp$outcome
 formula_temp <-
  as.formula(
   paste0(
    "scale(",
    outcome_temp,
    ")",
    "~",
    "scale(",
    predictor_temp,
    ")"))
 boot_temp <- boot(data = decathlon_results, boot_function,
R=500, formula = formula_temp)
 boot_temp_df <- as.data.table(boot_temp$t)
 boot_temp_df$predictor <- predictor_temp
 boot_temp_df$outcome <- outcome_temp
 boot_df <-
  rbind(
   boot_df,
   boot_temp_df
   )
 rm(orig_model_temp, predictor_temp, outcome_temp, formula_temp,
boot_temp, boot_temp_df)
}
```

11.5 Some minor formatting operations.

```
# Remove first row of dataframe #

boot_df <- boot_df[-1,]

# Rename 'V1' to 'estimate'

names(boot_df)[names(boot_df) == "V1"] <- "estimate"

# Create the grouping variable 'domain' #

boot_df$domain <-
    factor(
        gsub(
            "(_+[0-9]+[0-9])",
            "",
            boot_df$outcome),
         levels = c(
          "hundred_m",
    "long_jump",
    "shot_put",
    "high_jump",
    "four_h_metres",
    "one_ten_meters_hurdles",
    "discus_throw",
    "pole_vault",
    "javelin_throw",
    "one_five_k_meters"))

# Class conversion for 'predictor' #

boot_df$predictor <-
    factor(
     boot_df$predictor)
```

11.6 We generate factor variables with reverse-ordered levels. This is necessary because the coordinate system of the plots is flipped. Thus, factor levels also need to be "flipped".

```
boot_df$outcome_rev <-
 factor(
  boot_df$outcome,
  levels = rev(levels(boot_df$outcome)))

boot_df$predictor_rev <-
 factor(
  boot_df$predictor,
  levels = rev(levels(boot_df$predictor)))
```

Bootstrap ridgeline plot

12 Now the bootstrapped data are ready for plotting. We create a bootstrap ridgeline plot using ggplot2.

```
boot_ridgeplot <-
 ggplot(boot_df, aes(x = estimate, y = predictor_rev, fill =
outcome)) +
 geom_vline(xintercept = 0, lty = 1, linewidth = 1.5, color =
"#E73134") +
    geom_vline(xintercept = c(0.1, 0.3, 0.5), lty = "dotted",
linewidth = 0.6, color = "black") +
   geom_density_ridges(scale = 0.9, rel_min_height = 0.0001, color
= NA, alpha = .8)+
   labs(
    x = "Standardized regression coefficients",
    y = "",
    title = "") +
    theme_classic() +
 theme(
  legend.background = element_rect(fill = "white", colour = "black",
linewidth = 0.8),
  panel.grid.major.y = element_line(linewidth = 1, linetype =
"solid", color = "darkgrey"),
  axis.title.y = element_blank(),
  panel.border = element_rect(colour = "black", fill = NA, linewidth
= 1),
  panel.background = element_rect(fill = "white"),
  legend.position = "right",
  axis.text.y = text_settings,
        axis.text.x = text_settings,
        axis.title.x = text_settings,
        legend.text = text_settings,
  legend.title = text_settings) +
    scale_fill_manual(
        values = brewer.pal(length(levels(boot_df$outcome)), name =
"Paired"),
        name = "Outcome",
        labels =
          c(
    "hundred_m" = "100 meters",
    "long_jump" = "Long jump",
    "shot_put" = "Shot put",
    "high_jump" = "High jump",
    "four_h_metres" = "400 metres",
    "one_ten_meters_hurdles" = "110 meters hurdles",
    "discus_throw" = "Discus throw",
    "pole_vault" = "Pole vault",
    "javelin_throw" = "Javelin throw",
```

```
     "one_five_k_meters" = "1500 meters")) +
     scale_y_discrete(
      labels=c(
       "hematocrit" = "Hematocrit",
          "hemoglobin" = "Hemoglobin",
          "rb_cell" = "Red blood cell count",
          "iron" = "Iron",
          "transferrin" = "Transferrin",
          "ferritin" = "Ferritin",
          "haptoglobin" = "Haptoglobin")) +
     scale_x_continuous(breaks = c(-0.1, 0, 0.1, 0.2, 0.3, 0.4, 0.5,
 0.6))
```

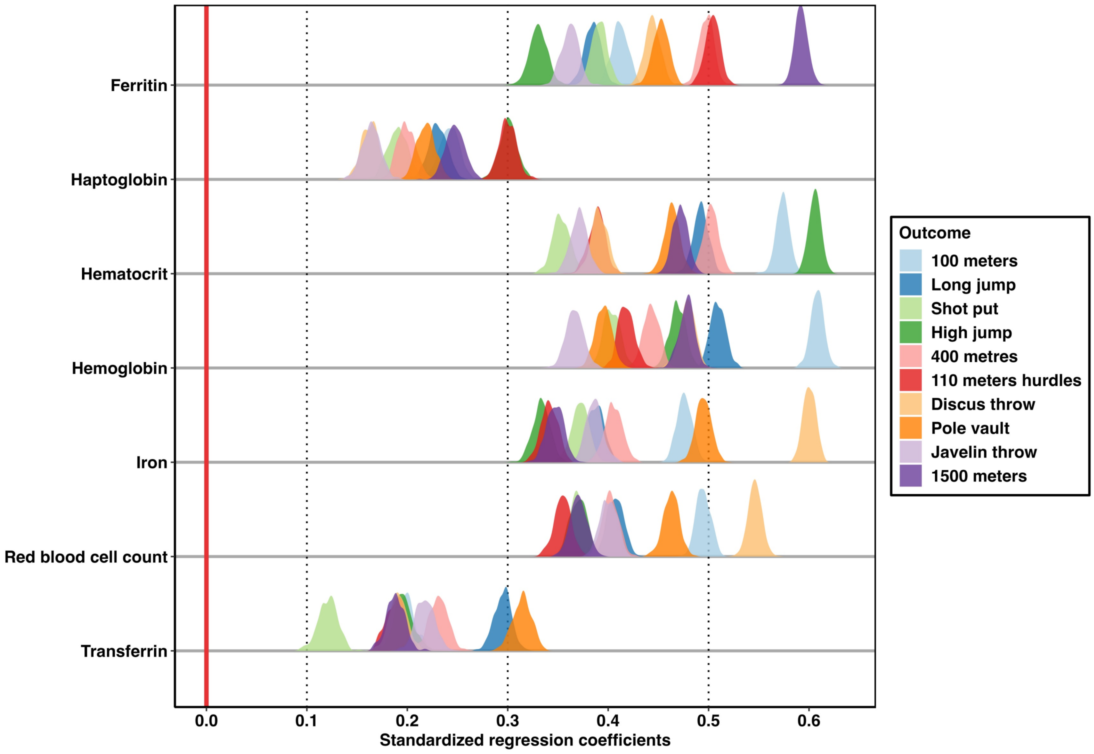

Bootstrap ridgeline plot, displaying miniature density plots of 500 parameter estimates for each of the 70 decathlon regression models.

Bootstrap violin plot

13 To address the issue of overlapping density plots, we developed an alternative representation of the distributions of bootstrapped beta coefficients by making use of the violin plot. To create a bootstrap violin plot, the bootstrap dataset from before can be used without modifications.

```
boot_violinplot <-
 ggplot(boot_df, aes(x = estimate, y = predictor_rev, fill =
outcome)) +
```

```
 geom_vline(xintercept = 0, lty = 1, linewidth = 1.5, color =
"#E73134") +
    geom_vline(xintercept = c(0.1, 0.3, 0.5), lty = "dotted",
linewidth = 0.6, color = "black") +
   geom_violin(
    color = NA,
    scale = 3,
    alpha = 1,
    position = position_dodge(width = 0.5))+
   xlab("Standardized regression coefficients") +
    ylab("") +
    ggtitle("") +
    theme_classic() +
 theme(
  legend.background = element_rect(fill = "white", colour = "black",
linewidth = 0.8),
  panel.grid.major.y = element_line(linewidth = 1, linetype =
"solid", color = "darkgrey"),
  axis.title.y = element_blank(),
  panel.border = element_rect(colour = "black", fill = NA, linewidth
= 1),
  panel.background = element_rect(fill = "white"),
  legend.position = "right",
  axis.text.y = text_settings,
        axis.text.x = text_settings,
        axis.title.x = text_settings,
        legend.text = text_settings,
  legend.title = text_settings) +
    scale_fill_manual(
        values = brewer.pal(length(levels(boot_df$outcome)), name =
"Paired"),
        name = "Outcome",
        labels =
          c(
    "hundred_m" = "100 meters",
    "long_jump" = "Long jump",
    "shot_put" = "Shot put",
    "high_jump" = "High jump",
    "four_h_metres" = "400 metres",
    "one_ten_meters_hurdles" = "110 meters hurdles",
    "discus_throw" = "Discus throw",
    "pole_vault" = "Pole vault",
    "javelin_throw" = "Javelin throw",
    "one_five_k_meters" = "1500 meters")) +
    scale_y_discrete(
     labels=c(
      "hematocrit" = "Hematocrit",
         "hemoglobin" = "Hemoglobin",
         "rb_cell" = "Red blood cell count",
         "iron" = "Iron",
```

```
        "transferrin" = "Transferrin",
        "ferritin" = "Ferritin",
        "haptoglobin" = "Haptoglobin")) +
    scale_x_continuous(breaks = c(-0.1, 0, 0.1, 0.2, 0.3, 0.4, 0.5,
0.6))
```

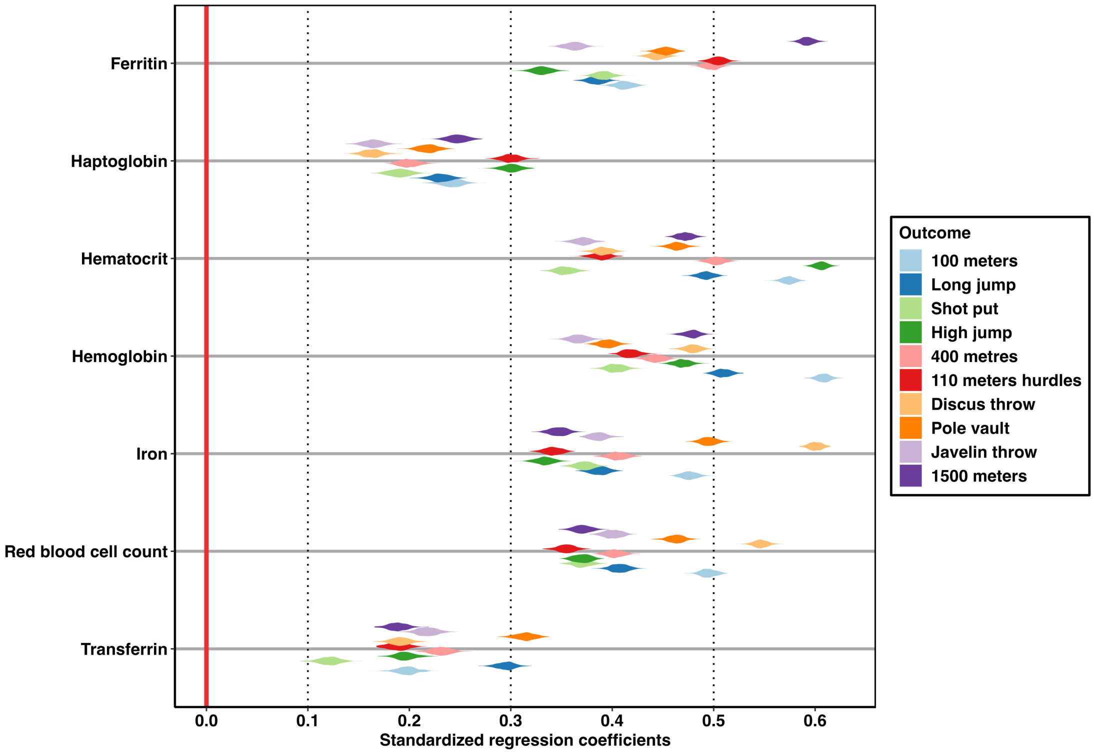

Bootstrap violin plot, displaying miniature density plots of 500 parameter estimates for each of the 70 decathlon regression models.
